# Comparison of three malignancy risk indices and CA-125 in the preoperative evaluation of patients with pelvic masses

**DOI:** 10.1186/1756-0500-4-206

**Published:** 2011-06-20

**Authors:** Zinatossadat Bouzari, Shahla Yazdani, Mahmoud Haji Ahmadi, Shahnaz Barat, Ziba Shirkhani Kelagar, Maryam Javadian Kutenaie, Nargeuss Abbaszade, Fateme Khajat

**Affiliations:** 1 Assistant professor, Department of OB&GYN of Babol University of Medical Sciences, Babol, Iran; 2 Member of stem cell researcher center of Babol University of Medical Sciences, Babol, Iran; 3 Assistant professor, Department of OB&GYN, Babol University of Medical Sciences, Babol, Iran; 4 Statistician, Babol University of Medical Sciences, Babol, Iran; 5 Assistant professor, Department of OB&GYN, Babol University of Medical Sciences, Babol, Iran; 6 Statistician, Babol University of Medical Sciences, Babol, Iran; 7 Medical student of Babol University of Medical Sciences, Babol, Iran; 8 B&GYN, Babol University of Medical Sciences, Babol, Iran

## Abstract

**Background:**

Patients with pelvic mass are the most referred patients to gynecologist. The aim of this study was to evaluate the ability of three malignancy risk indices (RMI 1, RMI 2 and RMI 3) and CA-125 to discriminate a benign from a malignant pelvic mass in our region (North of Iran).

**Methods:**

This retrospective study was performed on 182 women with pelvic masses referred to Yahyanejad Hospital from 2007 to 2009. Ultrasound scans were scored as one point for each of the following characteristics: multilocular cyst, solid areas, intra-abdominal metastases, ascites, and bilateral lesions. For each patient a total ultrasound score (U) was calculated. The difference of the three RMI was based on the allocation of the U and M scores. The sensitivity, specificity, positive predictive values (PPV) and negative predictive values (NPV) of level of serum CA-125, the RMI 1, 2 and 3 were compared.

**Results:**

Mean age of the patients was 39.9 ± 9.3 years. Most of them were premenopausal (161 women or 88.4%). A significant linear trend for malignancy was found by increasing age, ultrasound score, and serum CA-125. The best performance of CA125 was at a cut-off 88 U/ml, with a sensitivity of 88%, a specificity of 97%, a positive predictive value of 84%, and a negative predictive value of 99%. RMI 1 and 3 at the optimal cut off point of 265 and RMI2 at the optimal cut off point of 355, had a sensitivity of 91%, specificity of 96%, a positive predictive value of 78%, and a negative predictive value of 99%.

**Conclusion:**

In our population we found that there is no statistically significant difference in the performance of three malignancy risk indices (RMI 1, RMI 2, and RMI 3) and CA125 in differentiating between benign and malignant pelvic masses.

## Background

Patients with pelvic mass are the most referred patients to gynecologist. [1]. Ovarian cancer is one of the pelvic masses, the second most common gynecologic malignancy, the fifth cause of death due to cancers, and has more mortality than the other gynecologist malignancies [2,3]. Most cases are diagnosed at high stage where prognosis is very poor [4,5]. Regarding differentiation of benign versus malignant pelvic masses before surgery was difficult, therefore, Jacob et al. developed a Risk of Malignancy Index (RMI) based on serum level of CA125, menopausal state and ultra sound findings[6]. The RMI has been adjusted by Tingulstad et al. [7] in 1996 (RMI2) and again modified in 1999 (RMI3) [8]. The RMI is a suitable index for evaluation of pelvic mass before surgeries and confirms previous studies indicating that RMI improves the discrimination between non malignant and malignant pelvic masses[5,9].

In many studies, cut off value of 200 for RMI1 is the best discrimination for benign and malignant pelvic masses because of its high sensitivity and specificity levels[5]. The aim of this study was to evaluate the ability of three malignancy risk indices (RMI 1, RMI 2 and RMI 3) and CA-125 to discriminate a benign from a malignant pelvic mass in our region.(North of Iran)

## Methods

This retrospective study included the records of 182 consecutive women with pelvic masses, who were admitted for laparotomy between 2007 and 2009, at Yahyanejad Gynaecological Unit after signing a consent form approved by the Research Ethics Committee of Babol Medical University. Preoperative serum levels of CA-125 were measured by ELISA (Germany Roche Kit in the same laboratory), an ultrasonographic evaluation of their pelvic mass using a 2-7 MHZ abdominal transducer (General Electric, America) and a 12 MHZ transvaginal probe. Postmenopausal status was defined as more than 1 year of amenorrhea or an age of more than 50 years in women who have had a hysterectomy. Then RMI 1, 2, and3 were calculated for each patient. In all patients underwent the laparotomy, the histological specimens were sent to the pathology laboratory for the histopathologic diagnosis.

### Calculation of RMI

Ultrasound scans were scored as one point for each of the following characteristics: multilocular cyst, solid areas, intra-abdominal metastases, ascites, and bilateral lesions. For each patient a total ultrasound score (U) was calculated. The difference of the three RMI is based on the allocation of the U and M scores.

RMI 1 = U ՠM ՠCA125; an ultrasound score of 0 considered as U = 0, a score of 1 considered as U = 1, and a score of ? 2 considered as U = 3. Premenopausal status considered as M = 1 and postmenopausal status considered as M = 3. The serum level of CA125 was used directly in the calculation[6].

RMI 2 = U ՠM ՠCA125; an ultrasound score of 0 or 1 considered as U = 1, and a score of ¿ 2 considered as U = 4. Premenopausal status considered as M = 1 and postmenopausal status considered as M = 4. The serum level of CA125 was used directly in the calculation[7].

RMI 3 = U ՠM ՠCA125; an ultrasound score of 0 or 1 considered as U = 1, and a score of ¿ 2 considered as U = 3. Premenopausal status considered as M = 1 and postmenopausal status considered as M = 3. The serum CA125 level was used directly in the calculation[8].

### Statistical analysis

All data was analysed by SPSS18. We used the T-Test, Pearson Chi-square and Mann-Whitney U. Receiver Operating Characteristics (ROC) Curve was plotted and the sensitivity, specificity, positive(PPV) and negative predictive values(NPV) were determined. The McNemar's test was used when testing differences in performances between RMI 1, RMI 2 and RMI 3. A probability value of P < 0.05 was considered to be statistically significant.

## Results

Mean age of the patients was 39.9 ± 9.3 years. Most of them were premenopausal (161 women or 88.4%). The distribution of age, menopausal status, ultrasound score (U) and serum CA-125 level in women with benign and malignant pelvic mass were described in Table 1. In univariate analysis, a significant linear trend for malignancy was found by increasing age, ultrasound score, and serum CA-125. Table 2 lists the histology results, indicating that 158(87.3%), 23(12.7%) and 1(0.5%) were benign, malignant and tuberculosis masses, respectively.

**Table 1:** The distribution of age, menopausal status, ultrasound score (U), serum CA 125 level in women with benign and malignant pelvic masses

Parameter	Benign (n = 158)	malignant (n = 23)	P-value
Age (mean)	38.7 ± 8.3	47.7 ± 12.5	0.003*
menopausal status (M)			
Premenopausal	145(91.8%)	16(69.9%)	0.002*
Post menopausal	13(8.2%)	7(30.4%)	
Ultrasound score (U)			
U = 0	45(28.5%)	0 (0%)	0.001*
U = 1	50(31.6%)	0(0%)	
U = 2-5	63(39.9%)	23(100%)	
SerumCA-125(median)(U/ml)	21	112	< 0.001*

*P <0.05

**Table 2:** Definitive histopathological diagnosis of adnexal masses

Diagnosis	n	%
**Ovarian cancer**		
Stage I	8	4.3
Stage II	1	0.5
Stage III	11	6.0
Stage IV	3	1.6
**Total malignant tumors**	23	12.6
Metastatic tumor	2	1.1
Granulosa cells tumor	2	1.1
Serous papillary adenocarcinoma	9	4.9
dysgerminoma	1	0.5
Clear cell adenocarcinoma	1	0.5
Serous cystadenoma	1	0.5
Endometrioma	37	20.3
Dermoid cyst	29	15.9
hemorrhagic cyst	10	5.5
Paratubal cyst	21	11.5
Follicular simple cyst	10	5.5
Leiomyoma	10	5.5
Corpus luteum cyst	18	9.9
inclusion cyst	2	1.1
**Borderline Tumors**		
Mucinous adenocarcinoma	6	3.2
Serous adenocarcinoma	2	1.1
**Infective conditions**		
Tuberculosis mass	1	0.5

**Figure 1:**
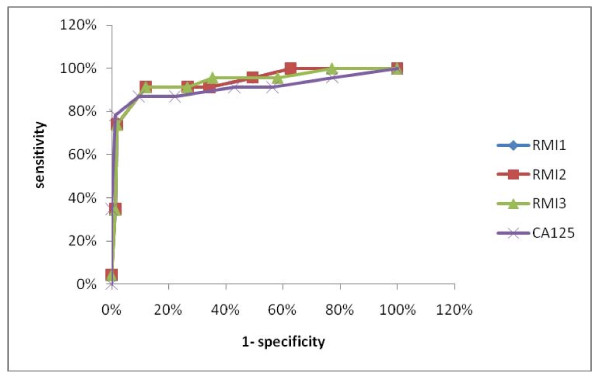
**ROC curve showing the relationship between specificity and sensitivity for RMI 1, RMI 2, RMI 3 and CA125 in differentiating between benign and malignant pelvic masses**.

The performance of RMI 1, RMI 2, RMI 3, and CA-125 at different cutoff levels are presented in Table 3. A direct comparison of the three RMI indices showed that there were not any significant difference between RMI1 and 3 at a cutoff level of 265 and RMI2 at a cutoff level of 355 (p > 0.05). The performance of RMI 1, RMI 2, RMI 3 and CA125 are presented in receiver operator characteristic curves (Figure 1). The detail of false positive and false negative cases based on the cut-off level criteria of RMI 1, 2, 3 and CA125 according to their histology are shown in Table 4.

**Table 3:** Sensitivity, specificity, positive, negative predictive values of three RMI and CA125

Methods	Sensitivity (%)	Specificity (%)	PPV (%)	NPV(%)
RMI 1&3 (cutoff: 200)	91	88	53	99
RMI 2 (cutoff: 200)	91	79	39	98
RMI 1&3 (cutoff: 265)	91	96	78	99
RMI 2 (cutoff: 355)	91	96	78	99
CA-125 (cut-off: 88 U/mL)	87	97	84	99

**Table 4:** False-positive cases and false-negative cases of three malignancy risk indices RMI 1, 2, 3 and CA125

	RMI 1 (cutoff: 265)	RMI 2 (cutoff: 355)	RMI 3 (cutoff: 265)	CA125 (cut off: 88)
False-positive cases				
endometriosis	5	5	5	3
Mucinous cystadenoma	1	1	1	1
False-negative cases				
granulosa cell tumor	1	1	1	1

## Discussion

Our study showed the usefulness of the CA125 in prereferral evaluation of patients with demonstrated pelvic masses. We were determined that the different serum level of CA 125 in benign and malignant pelvic mass is similar to other studies [1,10]. The best performance of CA125 in our study was at a cut-off 88 U/ml, with a sensitivity of 88%, a specificity of 97%, a positive predictive value of 84%, and a negative predictive value of 99%. Although, this good performance might lie on the distribution of tumor histology, which we showed in table 2.

The results of previous studies describe that many studies showed the best cut off point for RMI is 200[1,5-8,11]. However in this study, RMI 1 and 3 at the optimal cut off point of 265 and RMI2 at the optimal cut off point of 355, had a sensitivity of 91%, specificity of 96%, a positive predictive value of 78%, and a negative predictive value of 99%. Bailey et al. study on 182 women with pelvic masses indicated an RMI > 200 had a sensitivity of 88.5% for diagnosing invasive lesions[12] while Engelen et al. study on 302 women with pelvic mass indicated an RMI at a cut off point of 250 had a sensitivity of 88.2%, a specificity of 74.3%, a PPV of 71.3%, a NPV of 90% for diagnosing invasive lesions[13].

A systematic review study by Geomini P et al. in 2009, 116 diagnostic studies for adnexal malignancy was reviewed. The reported result showed that at the cut off point of 200, RMI has a sensitivity of 78% and specificity 87% for malignant mass diagnoses[14] which is similar to our report.

When RMIs cutoff was set at 265 or 355 and CA125 at 88 U/ml, all borderline tumors (mucinous adenocarcinoma and serous adenocarcinoma) and all stage I, were correctly identified prior to surgery, this increased the sensivity but not at the expense of increasing the false positive rate. Thus these cut offs are very useful at the peripheral hospitals and health centers to accelerate prompt referral of positive cases to tertiary hospitals. We showed that CA-125 at the cut-off 88 U/ml performed as well as the RMI 1 and 3 at a cut-off 265 and RMI 3 at a cut-off 355 in differentiating between benign and malignant pelvic masses.

## Conclusion

In our population we found that there is no statistically significant difference in the performance of three malignancy risk indices (RMI 1, RMI 2, and RMI 3) and CA125 in differentiating between benign and malignant pelvic masses.

## Competing interests

The authors declare that they have no competing interests.

## Authors' contributions

Each author has participated actively and sufficiently in this study. ZB conceived the idea and design of the study, interpretation of data, and drafted the manuscript. SHY conceived the idea and design of the study. MHJ and ZSH made substantial contribution to analysis and interpretation of data. SHB, MJK, NA, and FKH have made contribution to collecting of data and editing. Each author revised critically the manuscript and provided final approval of the version to be published.
